# Isolation and Characterization of Phenol-Degrading Psychrotolerant Yeasts

**DOI:** 10.1007/s11270-017-3391-8

**Published:** 2017-05-22

**Authors:** Natalia Filipowicz, Malwina Momotko, Grzegorz Boczkaj, Tomasz Pawlikowski, Marta Wanarska, Hubert Cieśliński

**Affiliations:** 1 Department of Molecular Biotechnology and Microbiology, Gdańsk University of Technology, Narutowicza 11/12, 80-233 Gdańsk, Poland; 2 Department of Chemical and Process Engineering, Gdańsk University of Technology, Narutowicza 11/12, 80-233 Gdańsk, Poland; 3 Fermentum Mobile Sp. z o.o. [Ltd.], 20 Podwale Przedmiejskie, 80-824 Gdańsk, Poland

**Keywords:** Biodegradation, Phenol, Psychrotolerant, Yeast, Peatland

## Abstract

In this study, the potential of selected psychrotolerant yeast strains for phenol biodegradation was studied. From 39 strains isolated from soil and water samples from Rucianka peat bog, three psychrotolerant yeast strains, A01_1_, B02_1_, and L01_2_, showed the ability to degrade phenol. The result shows that all three yeast strains could degrade phenol at 500 and 750 mg l^−1^ concentration, whereas strains A01_1_ and L01_2_ could degrade phenol at 1000 mg l^−1^ concentration. The time needed for degradation of each phenol concentration was no longer than 2 days. Strains A01_1_, B02_1_, and L01_2_ were identified based on 26S rDNA and ITS sequence analysis as belonging to species *Candida subhashii*, *Candida oregonensis*, and *Schizoblastosporion starkeyi-henricii*, respectively.

## Introduction

Phenol is one of the most widely distributed environmental pollutants which can be found mostly in wastewaters and industrial effluents. World annual production of this chemical compound is around 10 million tons, while 3.3 million tons are produced in European countries, 2.4 million tons in the USA, and 0.2 million tons in Russia (Federal State Statistics 2011). By the end of 2016, global phenol production is expected to increase by 2.1 million tons (ICIS 2016). Main sources of this hazardous compound are chemical industry, coal conversion processes, crude oil refineries, textile industry, and production of pesticides (Aleksieva et al. 2002; Boczkaj et al. 2016a, b, 2014). Also, it is an ingredient in many customer products like mouthwashes or antiseptic lotions. Phenol is present in food, especially in smoked, grilled, and fried meat, and is also detected in honey and coffee. Moreover, phenol is also formed during the decomposition of biomass and is released into the atmosphere during volcanic eruptions (ATSDR 2015). It is also formed during chemical reactions which lead to cloud formation. Some levels of phenols are also produced during UV irradiation (sunlight) from amino acids in plant’s hemicellulose and tyrosine transformation in digestive track (Davidson 1996; Tsuruta et al. 2000).

The important fact, in respect of environmental aspects, is that phenol is stable and resistant to self-degradation in water solutions. Moreover, it tends to accumulate in soil and groundwater and is able to move from soil to water. Phenol can be also detected in rainwater, surface water, and drinking water.

Due to its toxicity, phenol could become life-threatening to living organisms if proper precautions are not taken. When discharged into water, it can become a danger for fish life at low concentration 5–25 mg l^−1^ (Chung et al. 2003; Kibret et al. 2000; Kumar et al. 2005). Phenol at the concentration level of 1 g/1 kg of body weight is lethal to humans and animals but individual tolerance for this chemical compound can be higher (ATSDR 2015). Exposure to phenol occurs through breathing, skin contact, or ingestion.

The water or soil treatment of this toxic compound is focused on physical and chemical methods such as solvent extraction, adsorption, ion exchange, and advanced oxidation processes (AOPs) (preferably by ozonation) (Aksu 2001; Aksu et al. 1999; Banat et al. 2000; Gimeno et al. 2005; Hameed and Rahman 2008; Lin and Juang 2009; Rengaraj et al. 2002; Roostaei and Tezel 2004). For this purpose, adsorption of phenol on activated carbon is one of the most common methods. However, the major problems associated with this method are: (*i*) limited mechanical strength of activated carbon, (*ii*) high regeneration cost of this sorbent, and (*iii*) intraparticle resistance (Aravindhan et al. 2009; Siva Kumar et al. 2009). Besides, in all mentioned above methods, there is a risk of formation of hazardous by-products (Wang et al. 2000). In comparison with these methods, biological treatment of phenol has two major advantages: (*i*) low cost of mineralization process (Zümriye et al. 1999; Bux et al. 1999) and (*ii*) rare possibility of toxic secondary metabolite production (Wang et al. 2000). Hence, biodegradation of phenol is an attractive alternative for recently used physical and chemical methods of phenol removing. It could be carried out using biofilters for gaseous pollutions (García-Peña et al. 2008; Qi et al. 2002; Woertz et al. 2001), bioreactors for industrial effluents treatment (Jarvinen et al. 1994; Juárez-Ramírez et al. 2001), bioremediation techniques for contaminated waters (Puhakka et al. 1995) and soil (Cordova-Rosa et al. 2009), and natural attenuation for both water and soil samples (Broholm and Arvin 2000; Holder et al. 1999). Removal of phenol from contaminated soil and industrial effluents by bioremediation techniques can be achieved through aerobic biodegradation, by microorganisms utilizing this toxic aromatic compound as a sole source of carbon and energy. *Pseudomonas putida* has been the most extensively studied bacterium for aromatic compound biodegradation (Morasch et al. 2002; Reardon et al. 2002; Wang et al. 2008; You et al. 2013). *Alcaligenes eutrophus* (NRRL B 75940) (Hill et al. 1996), *Bacillus stearothermophilus* BR219 (Gurujeyalakshmi and Oriel 1989), and some bacterial strains belonging to *Acinetobacter* spp., *Citrobacter* spp., and *Shigella* spp. (Kafilzadeh et al. 2010) are also capable of degrading phenol at low concentration. Among yeast, *Candida tropicalis* is known to degrade high initial phenol concentration (beyond 1700 and 2000 mg l^−1^, respectively) in a relatively short period of time (Jiang et al. 2008; Yan et al. 2005). Moreover, *C. tropicalis* strains, for, e.g., strain CC1 (Galíndez-Mayer et al. 2008) and strain LB-L20 (Juárez-Ramírez et al. 2001), were successfully used in bioreactors for the removal of phenol. Yeast strains belonging to *Rhodotorula* spp. and *Trichosporon* spp. are also capable of phenol degradation (Kurtz and Crow 1997; Sampaio 1999). Advantages of using yeasts in bioremediation include rapid growth and ability to resist unfavorable environmental conditions (Yu and Wen 2005). Cold-adapted yeasts, with higher metabolic versatility than bacteria to biodegradation of petroleum hydrocarbon fractions (Margesin et al. 2003), are excellent candidates in studies on phenol bioremediation for contaminated soil and water in cold climatic regions.

On the other hand, most of the studied hydrocarbon-degrading microorganisms are mesophiles (Boroujeni et al. 2014; Chakraborty et al. 2010). However, the temperature of groundwater and soils in most regions of the world is lower than optimum of the growth temperature for mesophilic microorganisms (Bergauer et al. 2005). Therefore, in recent years, the interest in research on the use of psychrotolerant microorganisms to biodegradation of monoaromatic compounds has increased. Kotturi et al. (1991) showed the ability to degrade phenol by psychrophilic *P. putida* strain Q5. Margesin et al. (2004) described cold-tolerant *Arthrobacter* sp. strain AGG31 with the ability to use as sole source of carbon: phenol, *o*-cresol, *m*-cresol, catechol, hydroquinone, and salicylate. But there is still little known about psychrotolerant yeasts and their potential in aromatic compound biodegradation (Bergauer et al. 2005). Bergauer et al. (2005) described the ability to degrade monoaromatic compounds by cold-tolerant and psychrophilic yeast strains. Phenol and 18-phenol-related compounds were tested. None of 32 isolated strains were able to utilize any of the highly volatile monoaromatic compounds. Compounds which were easily utilized were phenol (100% of strains), hydroquinone (91% of strains), resorcinol (88% of strains), and catechol (75% of strains). However, in comparison to analogous research on bacteria, all compounds were utilized for yeast strains by growth at low concentration (50–200 mg l^−1^, depending on the compound).

Another significant factor, which should be considered, is the origin of yeast strain isolation. Microorganisms with the ability to degradation of phenol and other monoaromatic compounds are usually isolated from hydrocarbon contaminated sites caused by human activity, such as petroleum hydrocarbon-contaminated alpine soils (Margesin et al. 2005), sewage (Sivasubramanian and Namasivayam 2015; Wang et al. 2012), activated sludge (Jiang et al. 2008; Yan et al. 2005), oil-shale mine and railway area (Bergauer et al. 2005), oil refinery wastewaters (Rocha et al. 2007), or wastewater from coking plants (Karimi et al. 2016). Some investigations about ability of microorganisms from natural sources to aromatic compounds degradation were made (Kotturi et al. 1991; Margesin et al. 2004; Wang et al. 2008). Presumably, screening of microorganisms with potential for phenol and its derivatives biodegradation from other natural sources can be an alternative solution. In this study, yeast strains were isolated from peatland. Peatlands are rich in polyphenols and phenol derivatives which are products of lignocellulosic biomass decomposition (Thormann et al. 2007). Furthermore, it has been shown that yeast species which are involved in the process of biomass decomposition are also able to degrade simple and complex sugars, selected sugar alcohols, organic acids, and glycosides (Thormann and Rice 2007). Hence, we assumed that the soil and water samples from peat bogs could be an attractive source of microorganisms, especially psychrotolerant yeast species which are capable of biodegrading this class of monoaromatic compounds. However, there is still a lack of information about studies on yeasts isolated from peatlands with the ability to degrade phenol and its derivatives (Thormann et al. 2007).

Therefore, the purpose of this study was to isolate and identify psychrotolerant yeast strains from water and soil samples derived from Rucianka peatland, which are capable of utilizing phenol (at a wide range of concentration 500–2000 mg l^−1^) as the sole source of carbon.

## Materials and Methods

### Sample Collection

Soil and water samples were collected from Rucianka raised bog, located in northern-east Poland (54° 15′ 22″ N, 19° 44′ 14″) on January 5 and on April 7, 2015. Rucianka bog covers an area of 240 ha. It was formed during Vistulian glaciation and was filled by a lake. The bog was formed on sandy deposition and consists of two materials: fen and sphagnum peat. The exploration of peat started before Second World War, and by now, 60% of bog area consists of peat mine (Pawłat 1996).

Sterile 50-ml conical centrifuge tubes (Sarstedt, AG & Co., Germany) were used to collect samples. Soil samples were collected from 30 locations and each sample was collected only once from each place from depth about 5 cm. Water samples were collected from 19 locations such as drainage ditches, pipelines, and puddles, and each sample was collected only once from each place from depth about 2 cm. All samples were transported straight after a collection to the laboratory in the ice box to maintain the temperature around 4–8 °C.

### Isolation of Yeast Strains

One gram of each soil sample was transferred to sterile 50-ml conical centrifuge tube and mix vigorously with 9-ml sterile 0.9% saline solution for 10 s. After soil sedimentation, 100 μl of each sample was collected from 2 to 3 cm under water meniscus and spread with a sterile glass rod on YPD plates supplemented with chloramphenicol (stock solution = 34 mg ml^−1^) and ampicillin (stock solution = 100 mg ml^−1^). For water samples, 100 μl of each sample was transferred directly on YPD plate and spread with sterile glass rod. All plates were incubated at 18 °C for 6 days. After this period, colonies which differed from the other by morphological appearance (color, size, and shape of the colony) and microscopic examination were three times streak plating on YPD plates and incubated at 18 °C for 6 days to obtain a pure colony. Strain’s ability to grow on different temperature (4, 18, 30, and 37 °C) was tested.

Yeast extract peptone dextrose (YPD) contained (per liter) glucose 20 g, yeast extract 10 g, casein peptone 20 g, and bacteriological agar 20 g supplemented with 1 ml of each antibiotic: chloramphenicol (stock solution = 34 mg ml^−1^) and ampicillin (stock solution = 100 mg ml^−1^).

### Assimilation Tests—Screening for Yeast Strain Capable of Utilizing Phenol

Screening for yeast strains capable of utilizing phenol was done using a slant culture method (Middelhoven et al. 1991) with some modifications. Phenol was a sole source of carbon for yeast growth and the agar slants inoculated with tested yeast strains were incubated at 18 °C. If tested yeast strain was capable of utilizing phenol, a growth of yeast appeared on a slant surface. The growth intensity of tested strain on a slant surface depended on the toxicity of phenol towards this strain and the efficiency of utilization of phenol by this strain. Criteria used for evaluation the slant culture method were (++) for an intensive growth of yeast strain without inhibition zone, (+) for a weak growth of yeast strain with small inhibition zone (5–7 mm), and (−) for no growth or weak growth of yeast strain with inhibition zone >10 mm.

### Phenol Biodegradation

The first step of an experiment was a preparation of inoculation for each yeast isolate selected from assimilation test. This was achieved by inoculating 3-ml YPD medium with the single colony of examined yeast strain from actively growing YPD plate. For all yeast isolates, test tubes were incubated at 18 °C on the rotary shaker at the speed 170 rpm for 1 day. After this period, 80 μl of each yeast culture was transferred to 20 ml of fresh YPD medium in 200-ml Erlenmeyer flasks. Flasks were incubated at 18 °C on the rotary shaker at the speed 170 rpm for 3 days. Next, yeast cells were harvested and used as an inoculum. In all the experiments, 5% of subculture (1 ml) was inoculated into 20 ml of mineral salt medium (MSM) (Yan et al. 2005) in 200-ml Erlenmeyer flasks, supplemented with phenol at concentration 500, 750, 1000, 1500, and 2000 mg l^−1^, respectively. Mineral salt medium contained (per liter) (NH_4_)_2_SO_4_ 0.4 g, K_2_HPO_4_ 0.4 g, KH_2_PO_4_ 0.2 g, NaCl 0.1 g, MgSO_4_ 0.1 g, MnSO_4_·H_2_O 0.01 g, Fe_2_(SO_4_)_3_·nH_2_O 0.01 g, Na_2_MoO_4_·2H_2_O 0.01 g, and casein peptone 0.25 g, supplemented with 1 ml of each antibiotics: chloramphenicol (stock solution = 34 mg ml^−1^) and ampicillin (stock solution = 100 mg ml^−1^). The pH of the medium was adjusted to 6.0. At the start of incubation, OD of cultures were 0.2–0.3. All flasks were incubated at 18 °C on a rotary shaker at the speed 170 rpm for 5 days.

### Analytical Methods

The optical density of growing yeast cultures was measured spectrophotometrically by measuring the absorbance at wavelength 600 nm (Feng et al. 2002) using 96-well plate VIS 96/F-PS (Eppendorf) and Eppendorf PlateReader AF2200. One hundred microliters of each yeast culture was placed on a well plate. For every well, five measurements were done according to parameters: type, xy; size, 3 × 3; and border, 1350 μm.

### Process Control of Phenol Concentration During Biodegradation

To measure the concentration of phenol during biodegradation, simultaneously during optical density measurements, samples of suspended cultures were collected and centrifuged at 10,000 rpm for 10 min. One milliliter of each sample supernatant was placed in a 2-ml glass vial and 25 μl of 4-chlorophenol internal standard (IS) solution was added. The concentration of phenol in cell-free supernatant samples was determined by gas chromatography with a flame ionization detector (Clarus 580 GC, PerkinElmer, USA). An internal standard method was used. Detailed conditions of analysis are listed below.

- Column: 60.0 m × 0.32 mm (ID) × 1.8 μm (DB-624) (Agilent, USA)
- Carrier gas: nitrogen
- Flow rate (a constant flow mode): 2.0 ml min^−1^.
- The temperature program: initial temperature 60 °C, then a constant rate of 10 °C min^−1^ to final temperature 250 °C and then isothermally for 10 min
- Temperature of injector and detector (FID): 275 °C
- Sample injection: by autosampler, injection volume 2.0 ml, in splitless mode (for 1 min and after split mode 25:1)

### Identification of Yeast Strains by Sequencing of the D1/D2 Region and ITS Region

For genomic DNA isolation from selected yeast strains, one colony of each strain was used to inoculate 4-ml sterile YPD medium. All yeast cultures were grown at 18 °C on a rotary shaker at the speed 170 rpm for 4 days. Afterward, each yeast culture was harvested, and the cell pellets were used for genomic DNA isolation with ExtractMe DNA yeast kit according to manufacturer’s instructions (Blirt S.A., Poland).

For yeast identification, two different genomic DNA regions were amplified by PCR, using DNA polymerase *Pwo* HyperNova (Blirt S.A., Poland). First was D1/D2 rDNA region which was amplified using the following primer pair: forward NL-1 (5′-GCATATCAATAAGCGGAGGAAAAG-3′) and reverse NL-4 (5′-GGTCCGTGTTTCAAGACGG-3′) (Hesham et al. 2006).

PCR was performed for 30 cycles including an initial denaturation at 95 °C for 60 s, subsequent denaturation at 95 °C for 30 s, annealing at 59 °C for 30 s, and extension at 72 °C for 45 s followed by final extension at 72 °C for 5 min and holding at 4 °C.

The second region was 5.8S-ITS rDNA region which was amplified using the following primer pair: forward ITS-1 (5′-TCCGTAGGTGAACCTGCGG-3′) and reverse ITS-4 (5′-TCCTCCGCTTATTGATATGC-3′) (Kurtzman and Robnett 1998).

PCR was performed for 30 cycles including an initial denaturation at 95 °C for 60 s, subsequent denaturation at 95 °C for 30 s, annealing at 52 °C for 60 s, and extension at 72 °C for 45 s followed by final extension at 72 °C for 5 min and holding at 4 °C.

Next, PCR amplification products were purified using ExtractMe DNA Clean-up Kit (Blirt S.A., Poland) according to manufacturer’s instructions. PCR products were sent to Genomed S.A., Poland and directly sequenced using 3730xl DNA Analyzer (Applied Biosystems, Life Technologies). PCR primers NL-1 and NL-4 of the D1/D2 26S rRNA gene and ITS1 and ITS4 of 5.8S-ITS region were used in the sequencing reactions to reading both DNA strands. The resulting DNA sequences were compared to the appropriate DNA sequences of the GenBank database using the basic local alignment search tool (BLAST) at the National Center for Biotechnology Information (http://www.ncbi.nlm.nih.gov/BLAST/). Phylogenetic trees for studied yeast strains were constructed with MEGA version 6.0 using a neighbor-joining algorithm and bootstrap analysis (1000) (Tamura et al. 2013).

## Results and Discussion

### Isolation of Yeast Strains

A total of 39 psychrotolerant yeasts were isolated from water and soil samples of Rucianka raised bog. Twenty-one isolates were obtained from water samples and 18 isolates were isolated from soil samples. For each isolate, a morphology of colony and cell morphology were studied. The pure cultures of all isolates were deposited in yeast strain collection of the Department of Molecular Biotechnology and Microbiology, Gdańsk University of Technology. All of the isolates were cryoconserved and kept under −80 °C.

### Isolation of Phenol Utilizing Yeasts

After 1 week of slant incubation, growth density was estimated. For 6 from 39 examined yeast strains, intensive growth on the whole surface of slant was observed. Moreover, for these strains, there was no zone of growth inhibition on slants and turbidity of water-phenol solution present above slant surface was observed. For 23 yeast strains, growth on slants was classified as weak. Intensity was low and zone of growth inhibition was 5–7 mm. For 10 yeast strains, no growth or growth of few single yeast colonies was observed. For those strains, the zone of inhibition was greater than 10 mm (Table 1).

**Table 1:** Growth intensity on slants

Strain number	Collection number	Growth intensity
A02_3_, A02_5_, B01_2_, F03_2_, G02_1_, J02_1_, M03_3_, P02_1_, S01_2_, S01_3_	373, 374, 375, 353, 376, 358, 366, 367, 377, 378	−
A03_1_, C02_1_, C02_2_, C03_1_, D01_1_, D01_4_, D01_5_, D02_1_, F03_1_, F03_4_, I02_2_, J02_2_, K01_1_, K01_2_, L01_1_, M03_1_, M03_2_, N02_1_, P03_1_, R01_1_, R01_2_, R01_3_, S01_1_	326, 328, 329, 379, 380, 381, 351, 350, 352, 382, 385, 359, 360, 361, 362, 364, 365, 383, 368, 369, 370, 371, 384	+
A01_1_, B02_1_, I02_1_, L01_2_, S01_4_, A02_1_	323, 324, 357, 363, 372, 325	++

Strain number: number assigned to pure culture after isolation from water or soil sample. Collection number: number assigned to strain (pure culture) deposited in the collection of Department of Molecular Biotechnology and Microbiology, Gdańsk University of Technology. Growth intensity: according to 1.2.3 in “Materials and Methods”

Based on this method, six strains were selected for further studies. These strains were resistant to toxic properties of phenol, and presumably, these strains were able to use phenol as a sole carbon source.

## Phenol Biodegradation—Results and Discussion

As mentioned above, most investigations are focused on phenol degradation by mesophilic microorganisms. Among them, bacteria are the most extensively examined. Bacteria are able to mineralize phenol at 100–1200 mg l^−1^ concentration (Polymenakou and Stephanou 2005) but the greater part of publications is focused on study of ability to use of phenol at 200 mg l^−1^ concentration. Also, several yeast strains were reported as capable of phenol biodegradation. The highest phenol concentration was degraded by *Candida maltosa* (1700 mg l^−1^ concentration) (Fialová et al. 2004) and *C. tropicalis* (up to 2000 mg l^−1^) (Yan et al. 2005). Therefore, for our selected psychrotolerant yeast strains, we decided to test their ability to degrade phenol at 500, 750, 1000, 1500, and 2000 mg l^−1^ concentration, respectively.

**Fig. 1:**
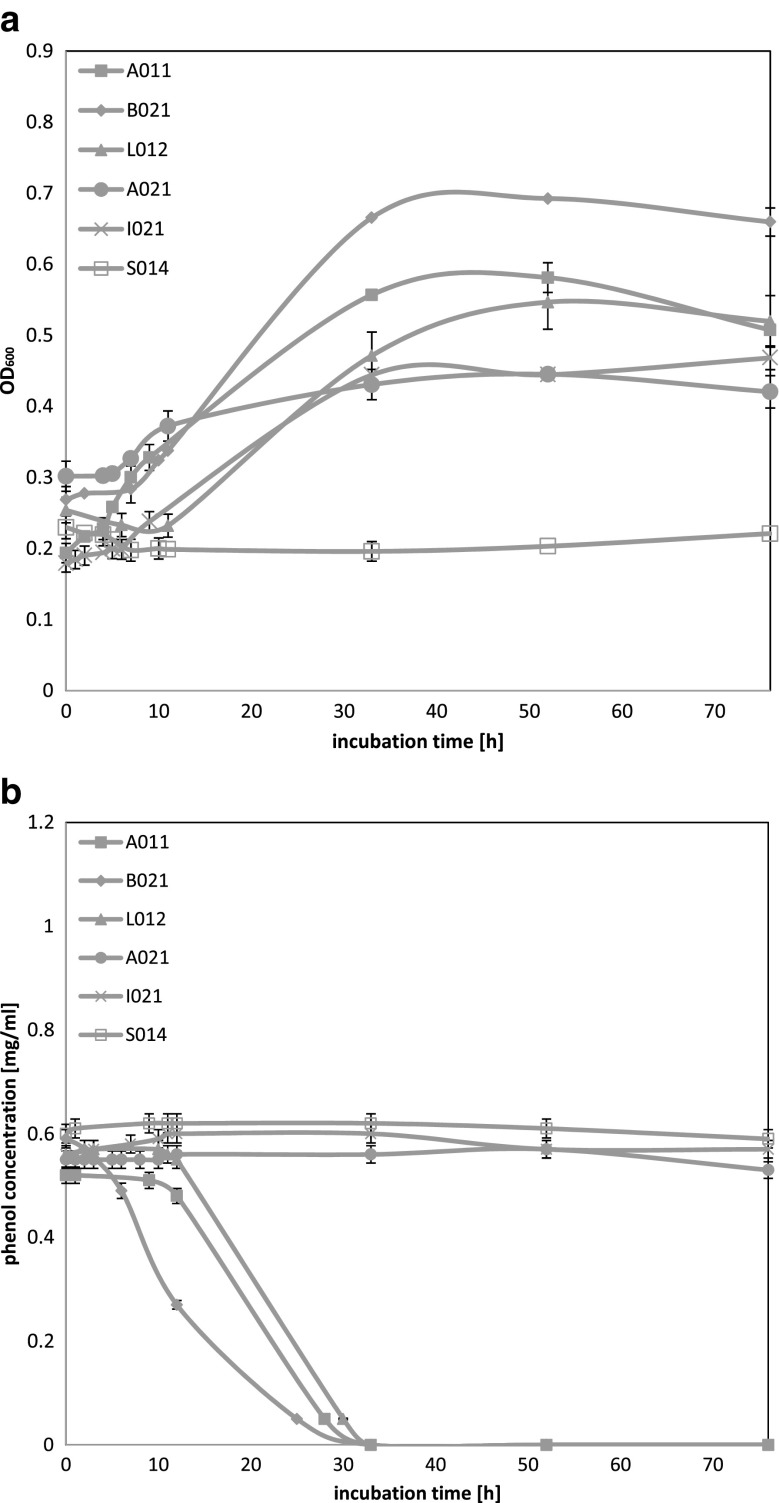
Growth curves for yeast strains in MSM medium supplemented with phenol at 500 mg l^−1^ concentration (**a**) and residual phenol concentration curves (**b**). Each strain has defined marker pointed out on figure

Figure 1a shows growth of selected six yeast strains during incubation time (76 h). For three of six examined strains, a significant increase of OD_600_ values and a decrease of residual phenol concentrations were observed. Strains A01_1_, B02_1_, and L01_2_ showed an increase in the values of OD_600_ between 10 and 48 h of culture incubation (logarithmic phase of growth). After this period, OD_600_ values remained stable on the same level or decreased slightly with time. Strains B02_1_ and L01_2_ exhibited an adaptation phase which lasted for around 10 h when for strain A01_1_ OD_600_ values started to increasing since of the beginning of its culture incubation. For strains A02_1_ and I02_1_, slightly increasing OD_600_ values were observed but it was not associated with decreasing of residual phenol concentration. For strain S01_4_, no connection between OD_600_ values and residual phenol concentration was observed and it is likely that phenol is toxic for this microorganism. Strains A02_1_, I02_1_, and S01_4_ were excluded from further studies.

Analysis of residual phenol concentration chart (Fig. 1b) leads to conclusions that strains A01_1_, B02_1_, and L01_2_ were able to reduce phenol from 500 mg l^−1^ concentration to below the detection limits in less than 30 h of the culture incubation. Reduction of phenol corresponded with increasing OD_600_ values. After utilization of phenol as a sole source of carbon, no significant changes of OD_600_ values were observed.

**Fig. 2:**
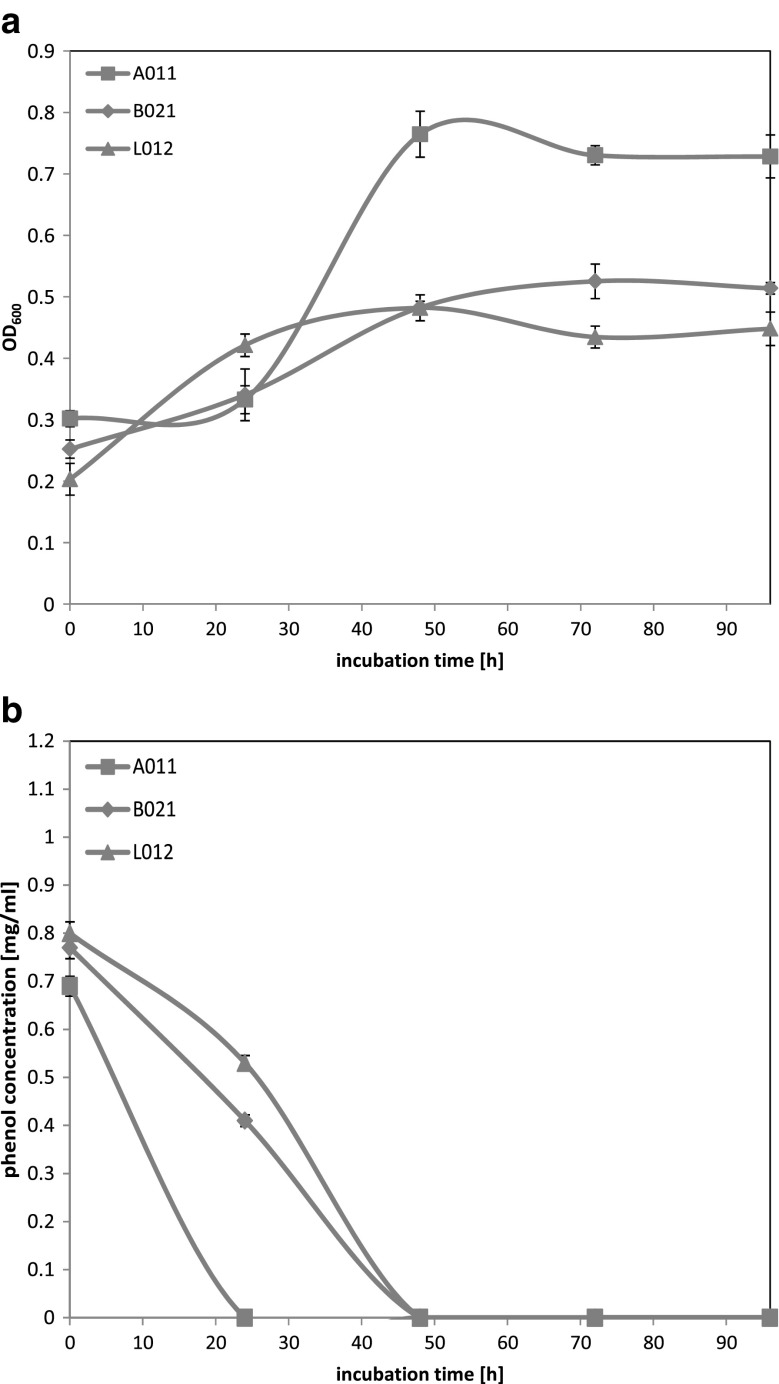
Growth curves for yeast strains in MSM medium supplemented with phenol at 750 mg l^−1^ concentration (**a**) and residual phenol concentration curves (**b**). Each strain has defined marker pointed out on figure

The next examined phenol concentration was 750 mg l^−1^. Only three strains, selected on 500 mg l^−1^ phenol concentration, were tested. Isolate A01_1_ exhibited an adaptation phase which lasted around 24 h, whereas, for strains B02_1_ and L01_2_, OD_600_ values started to increase directly after starting of the culture incubation (Fig. 2a). Moreover, during the culture incubation time, the largest increase of OD_600_ values was observed for strain A01_1_ (logarithmic growth phase between 24 and 48 h), whereas for strains B02_1_ and L01_2_, the increase of OD_600_ values was less significant and lower yeast biomasses were yielded. Highest OD_600_ values were reached around 48 h of culture cultivation for strains A01_1_ and L01_2_ and around 70 h for strain B02_1_. After this period, OD_600_ values remained stable on the same level or decreased slightly.

Chart of residual phenol concentration (Fig. 2b) reveals that all three strains were able to reduce phenol below detectable concentration. For isolate A01_1_, reduction of phenol followed after 24 h of incubation and for strains B02_1_ and L01_2_ after around 48 h, respectively. Isolates B02_1_ and L01_2_ used phenol from the beginning of incubation, what corresponded with changes of OD_600_ values. For strain A01_1_, the OD_600_ values increased faster and reached much higher levels, and along with these changes, phenol degradation was faster than for the other strains. In comparison to results obtained from previous chart of residual phenol concentration, for strain A01_1_, incubation time needed for degradation of phenol at 500 and 750 mg l^−1^ was similar, whereas for strains B02_1_ and L01_2_, reduction of phenol at 750 mg l^−1^ concentration below detectable level took twice as much time as for 500 mg l^−1^ concentration.

**Fig. 3:**
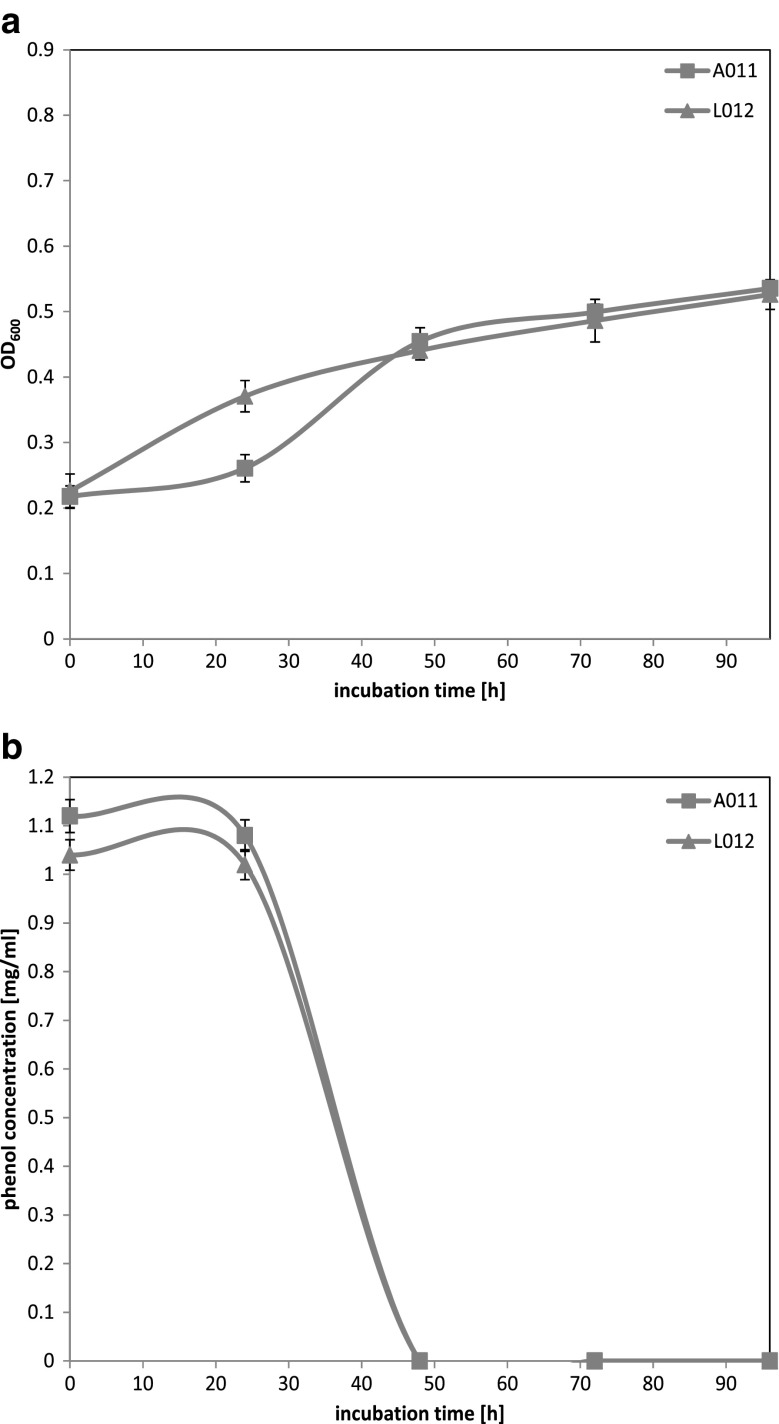
Growth curve for yeast strains in MSM medium supplemented with phenol at 1000 mg l^−1^ concentration (**a**) and residual phenol concentration curves (**b**). Each strain has defined marker pointed out on figure

The next tested phenol concentration was 1000 mg l^−1^. Only two strains, A01_1_ and L01_2_ (Fig. 3a), were able to grow in the presence of high concentration of phenol. For both strains, A01_1_ and L01_2_, OD_600_ values at the beginning and the end of the incubation, were comparable. For isolate A01_1_, similarly to 750 mg l^−1^ concentration, adaptation phase lasted for 24 h whereas for isolate L01_2_ OD_600_ values started to increase immediately after starting of incubation. Chart of residual phenol concentration (Fig. 3b) revealed that curves for each strain were similar. Both strains were able to reduce phenol concentration below the detectable level in around 48 h.

To sum up, the analysis of Figs. 1 and 2 leads to main conclusions. Three psychrotolerant yeast strains A01_1_, B02_1_, and L01_2_ exhibited an effective degradation of phenol at 500 and 750 mg l^−1^ concentration. Two strains (Fig. 3), A01_1_ and L01_2_, were able to grow in the presence of phenol at 1000 mg l^−1^ concentration. In all experiments, complete degradation of phenol was possible in around 2 days. For all analyzed yeast strains, higher initial phenol concentration required longer incubation time what could be associated with potential toxic phenol influence on this yeast metabolism. None of the selected strain was able to grow in the presence of phenol concentration (at 1500 or 2000 mg l^−1^, respectively). Presumably, phenol at these concentrations had a toxic influence on a growth rate of all examined strains. Besides, the production and accumulation of other intermediates of phenol degradation pathways may be responsible for decreasing cell mass yield and toxicity of phenol (Allsop et al. 1993).

Among psychrotolerant microorganism, some strains of *Rhodococcus* spp. were able to mineralize phenol in a concentration ranging from 1 to 12.5 mM (around 94–1176 mg l^−1^) (Margesin et al. 2005). Degradation was done within 5 days while higher concentration was done in 14 days. Considering yeast strains, 400 mg l^−1^ concentration was investigated (Bergauer et al. 2005). Moreover, Margesin et al. (2005) pointed out yeast isolates able to degrade phenol concentration as high as 15 mM (around 1412 mg l^−1^). Phenol up to 5 mM (around 470 mg l^−1^) was degraded within 3 days and 7.5 mM (around 705 mg l^−1^) within 7 days. The time which was needed to effectively degrade was longer than the results obtained for yeast strains presented in this study. Strains isolated from peat bog exhibited effective phenol degradation in less than 2 days. These comparisons should be considered only as an overview, because specific methodology of each investigation effect on the time and specific rate of phenol degradation.

### Identification of Yeast Strains

Appropriate PCR products (for ITS region or D1/D2 region) were purified, sequenced, and analyzed for nucleotide matching by BLAST (Nucleotide BLAST, high similar sequences—megablast).

**Fig. 4:**
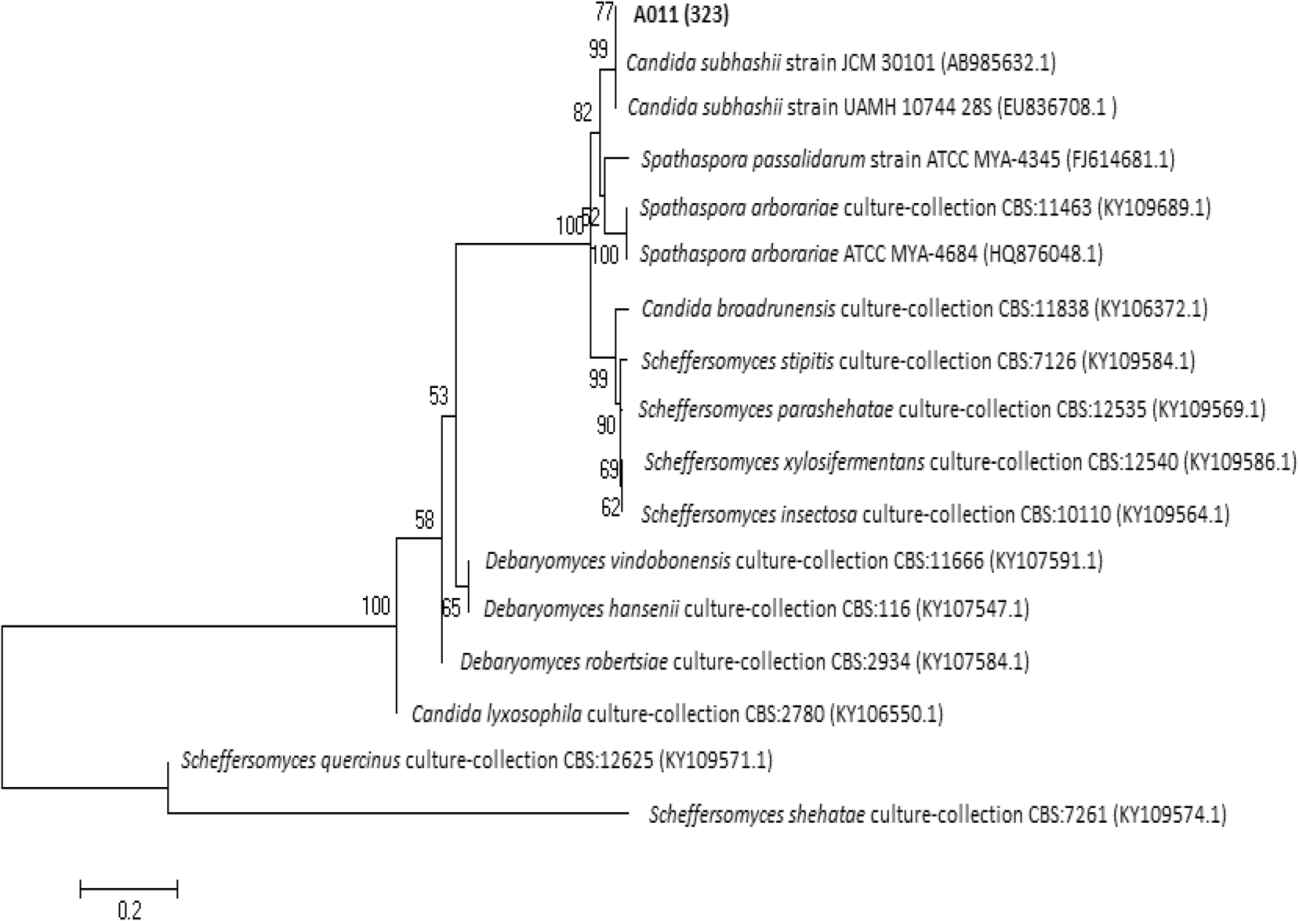
Phylogenetic tree based on the D1/D2 region of rDNA for strain A01_1_

For D1/D2 rDNA region of strain A01_1_ (613 bp fragment), the highest DNA sequence identity (99%) and cover (100%) was found with *Candida subhashii* strain UAMH 10744 (GenBank accession number EU836708.1). Strain UAMH 10744 is a type strain, which was deposited in the University of Alberta Microfungus Collection and Herbarium, Edmonton, Canada. It was also deposited in CBS-KNAW (Centraalbureau voor Schimmelcultures, Utrecht, the Netherland) as CBS 10753 and in Mycobank as MB 512099. For this *C. subhashii* strain, both conventional fermentation and assimilation tests in liquid were performed (Adam et al. 2009). Analysis of D1/D2 rDNA-based phylogenetic tree (Fig. 4) revealed that strain A01_1_ clustered not only with *C. subhashii* strain UAMH 10744 but also with *C. subhashii* strain ATY945 (AB985632.1). However, the characterization of strain ATY945 with biochemical tests was not done (Tanimura et al. 2015).

**Fig. 5:**
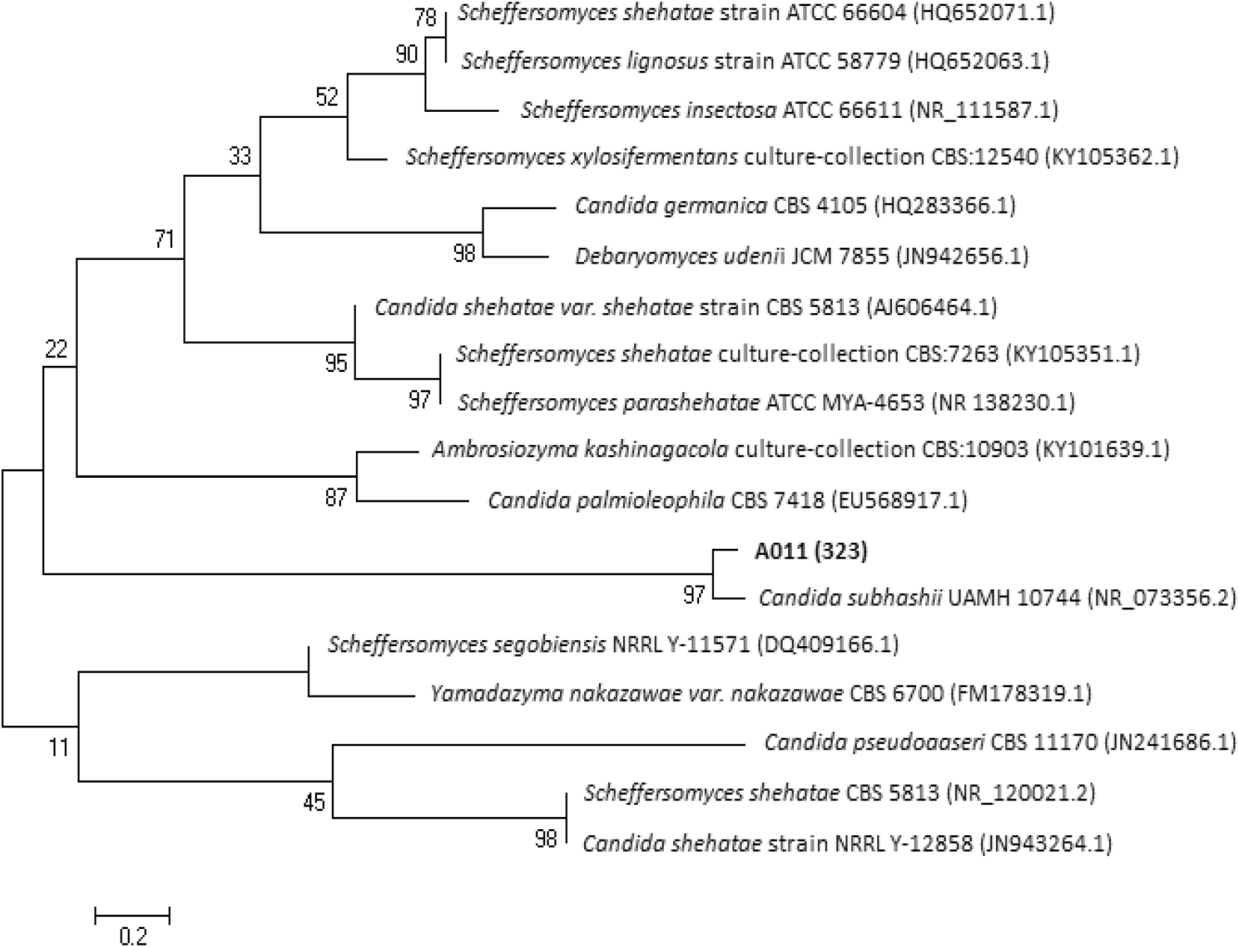
Phylogenetic tree based on the ITS region of rDNA for strain A01_1_

For analyzed ITS sequence (565 bp fragment), this strain showed highest DNA sequence identity (96%) and cover (100%) with *C. subhashii* type strain UAMH 10744 (NR_073356.2). Also a phylogenetic tree based on ITS sequences analysis shows that analyzed strain clustered with strain UAMH 10744 (Fig. 5).

To sum up, results obtained after above-mentioned analysis show that the strain A01_1_ might belong to *C. subhashii*. Although the distance and composition of trees branches and clusters led to the hypothesis that clustered might be connected with different yeast species than *Candida* spp. (Fig. 4 and Fig. 5). Cluster contained strain A01_1_ adjoined with major clusters gathered strains of different species. It was also suggested by Adam et al. (2009) where *C. subhashii* strain UAMH 10744 belonged to a different branch than other *Candida* sp. strains.

**Fig. 6:**
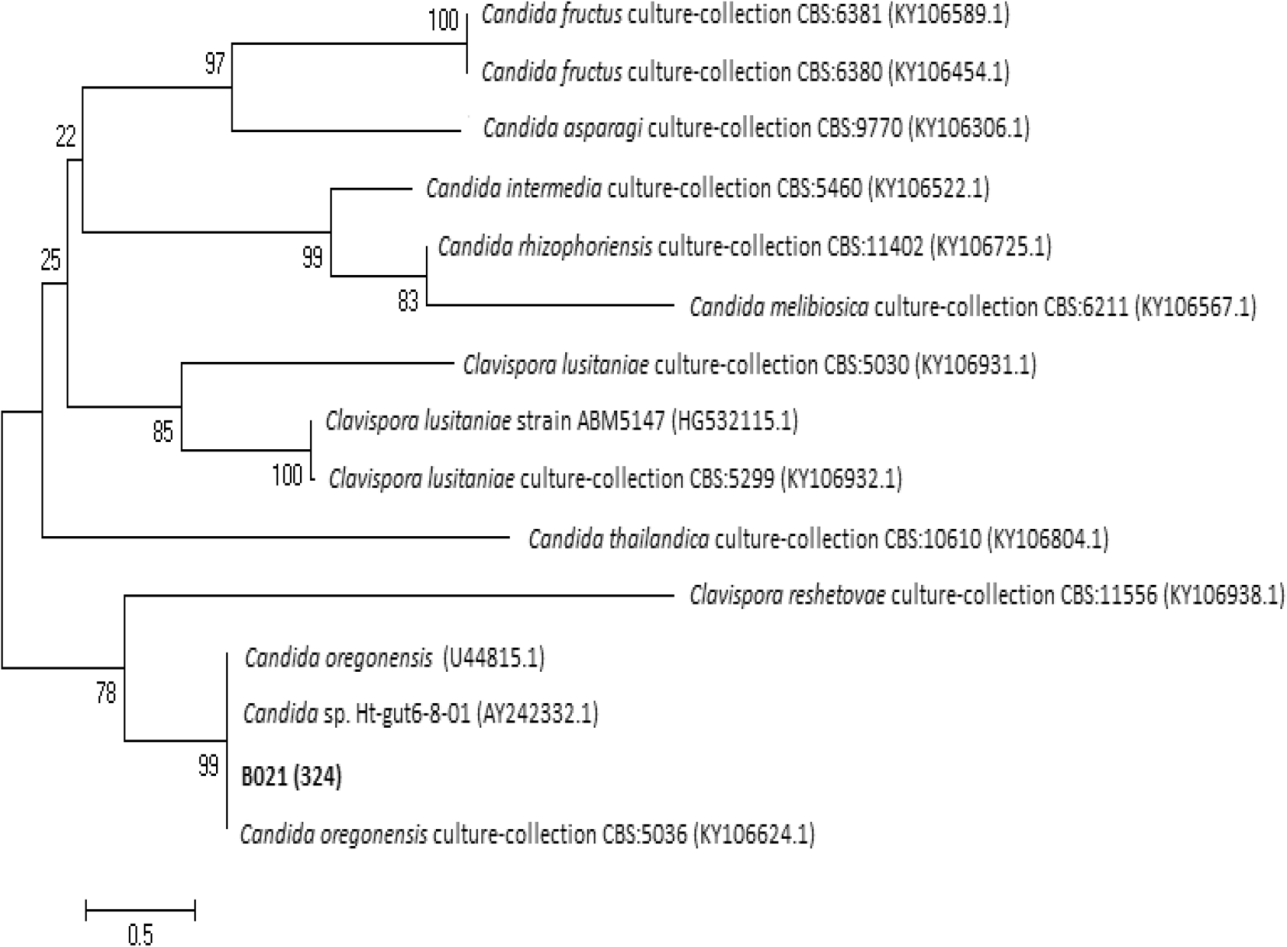
Phylogenetic tree based on the D1/D2 region of rDNA for strain B02_1_

For D1/D2 rDNA region of strain B02_1_ (558 bp fragment), the highest DNA sequence identity (100%) and cover (96%) was found with *Candida oregonensis* strain (KY106624.1). This strain was deposited in CBS-KNAW culture collection as CBS 5036. Phylogenetic tree based on D1/D2 region (Fig. 6) shows that selected strain clustered with strain *Candida* sp. Ht-gut 6-8-01 (AY242332.1) and *C. oregonensis* (U44815.1), where both sequences show 100% identity between each other. Identification of strain Ht-gut 6-8-01 based on D1/D2 loop sequence of the LSU rRNA gene. Only some of isolates from each LSU genotype were deposited in collections and morphological observations and metabolic tests were performed (Suh et al. 2004). In case of type strain deposited in GenBank as U44815.1, it was maintained in the Agricultural Research Service Culture Collection, National Center for Agricultural Utilization Research, Peionia, III (NRRL Y-5850) and was also deposited in CBS-KNAW as CBS 5036 (Kurtzman and Robnett 1997).

**Fig. 7:**
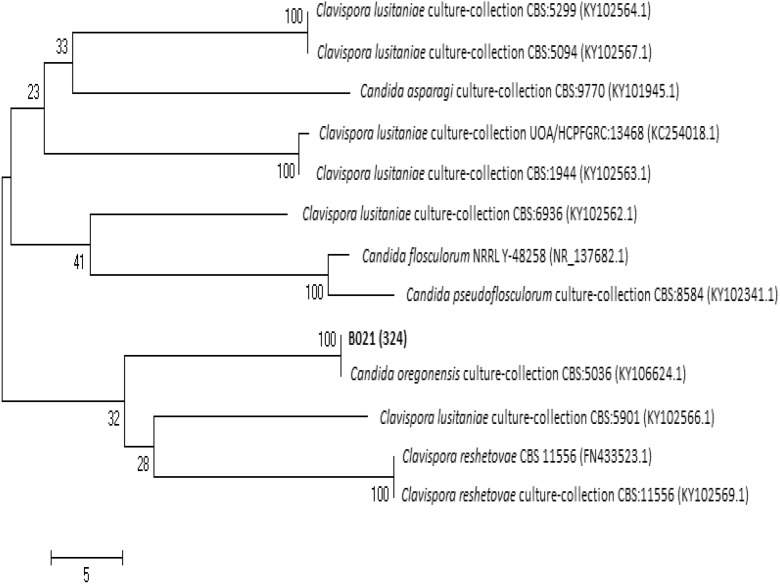
Phylogenetic tree based on the ITS region of rDNA for strain B02_1_

For ITS region (381 bp fragment), analyzed DNA sequence of strain showed 99% identity and 100% cover also with *C. oregonensis s*train CBS 5036. ITS region-based phylogenetic tree shows that selected strain clustered also with *C. oregonensis* strain CBS 5036 (Fig. 7).

According to these results, strain B02_1_ may be classified as *C. oregonensis*.

**Fig. 8:**
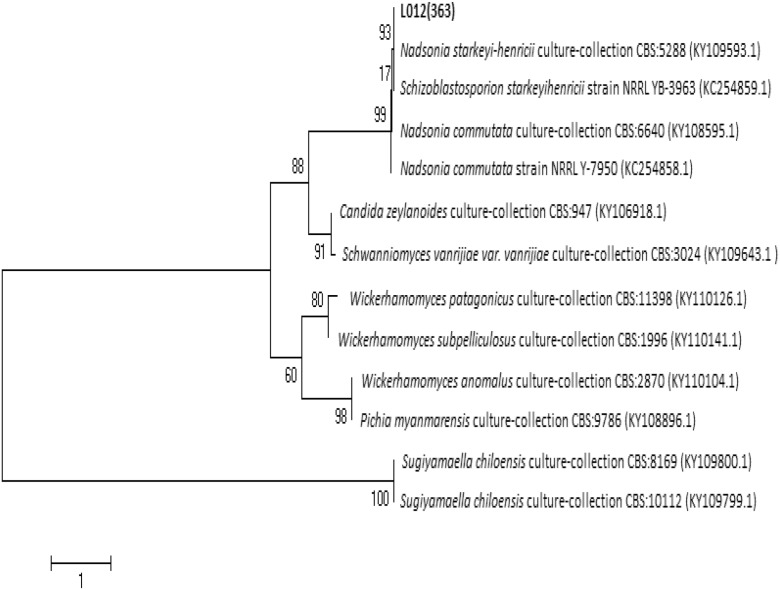
Phylogenetic tree based on the D1/D2 region of rDNA for strain L01_2_

For the D1/D2 rDNA region of strain L01_2_ (613 bp fragment), the highest DNA sequence identity (99%) and cover (100%) were found with *Nadsonia starkeyi-henricii* culture collection strain CBS 5288 (KY109593.1). For this yeast species, authority name is *N. starkeyi-henricii* and synonymic names are *Schizoblastosporion starkeyi-henricii* and *Schizoblastosporion starkeyihenricii* (Kurtzman and Robnett 2013). Phylogenetic tree generated from the analysis of other closely related D1/D2 rDNA nucleotide sequence revealed that this strain clustered with *S. starkeyi-henricii* strain NRRL YB-3963 (KC254859.1) and also above-mentioned *N. starkeyi-henricii* strain CBS 5288 (KY109593.1) (Fig. 8).

**Fig. 9:**
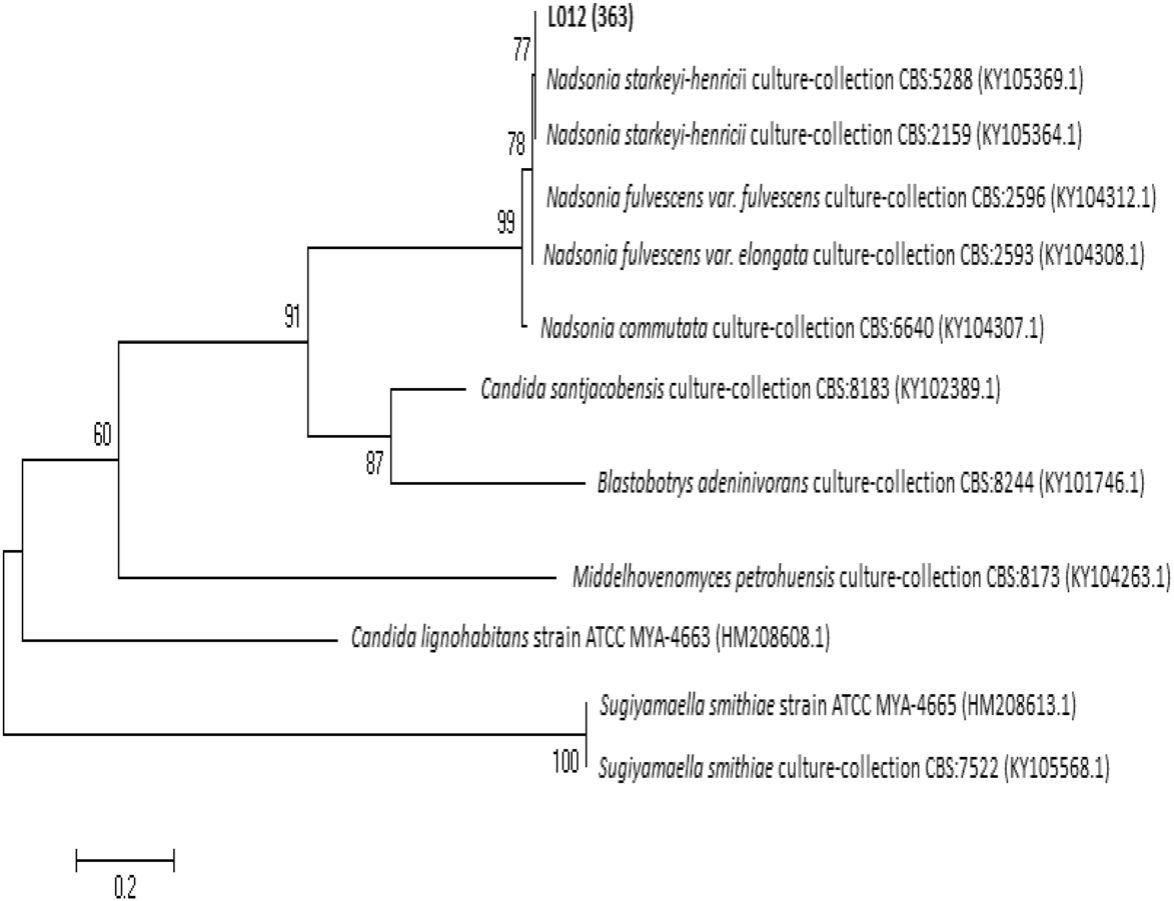
Phylogenetic tree based on the ITS region of rDNA for strain L01_2_

For ITS region BLAST analysis, the L01_2_ strain (571 bp fragment) shows 99% identity of DNA sequence with 99% of cover with analogous DNA sequence *N. starkeyi-henricii* culture collection strain CBS 5288 (KY109593.1), the same strain which revealed the highest DNA sequence identity for D1/D2 region DNA sequence analysis. ITS region-based phylogenetic tree shows that strain L01_2_ clustered with two *N. starkeyi-henricii* strains—strain CBS 5288 (KY109593.1) and CBS 2159 (KY105364.1) (Fig. 9).

To sum up, analysis of D1/D2 rDNA and ITS region led to the hypothesis that strain L01_2_ may be a member of species *S. starkeyi-henricii* (*N. starkeyi-henricii*). Moreover, reference strain of *S. starkeyi-henricii* (ATCC 24615) was also isolated from soil collected from a peat bog. Furthermore, this yeast genus appeared in multiple studies on peats and fens in Europe, Canada, Asia, and New Zealand and in most reports about peat bog microorganisms (Babjeva and Blagodatskaia 1971; Hong et al. 2006; Polyakova et al. 2001). It is considered as a rare genus with not fully classified taxonomy and isolated mainly on peatlands (Golubev and Pfeiffer 2014; Polyakova et al. 2001).

To date, there is no information about studies focused on the evaluation of the usefulness of strains belonging to *C. subhashii* (Adam et al. 2009; Fricova et al. 2010; Tanimura et al. 2015; Valach et al. 2011; Watanabe et al. 1998) and *C. oregonensis* (Dohet et al. 2016; Lou et al. 2014; Phaff and do Carmo-Sousa 1962, Rivera et al. 2007) to biodegradation of aromatic hydrocarbons. In this connection, some yeast strains (G28 and G38) belonging to species *S. starkeyi-henricii* exhibited a potential of assimilation of benzene derivative compounds. According to culture slant method, strain G28 assimilated phenol, catechol, resorcinol, hydroquinone, gentisic acid, and 3-hydroxybenzoic acid at 25 °C. Yeast strain G38 was able to degrade phenol at 10 °C based on yeast enrichment cultures on solid media (Middelhoven et al. 1992).

## Conclusions

In this study, the potential of psychrotolerant yeasts isolated from peatland to biodegrade phenol was studied for the first time. Phenol as the only carbon source was degraded by three yeast strains isolated from soil and water samples collected from Rucianka peat bog. Each strain was able to degrade phenol at 500 and 750 mg l^−1^ concentration and two strains at 1000 mg l^−1^ concentration in the mineral salt medium during incubation at 18 °C. All phenol concentrations were degraded below detectable levels in less than 48 h. None of the strains were able to degrade higher phenol concentration (1500 or 2000 mg l^−1^).

The data obtained in this study shows that the contribution of cold-adapted yeasts to biodegradation of phenol is underestimated. Most investigations on low temperatures are focused on bacteria, whereas knowledge about yeasts is limited. Moreover, according to literature, biodiversity of yeast isolated from peatlands has received little attention. This study pointed out a new direction to search of psychrotolerant yeasts with biodegradation of organic compound potential. Selected strains, especially strain L01_2_ and strain A01_1_, may be useful for examination of degradation for other monoaromatic toxic compounds, like BTEX group.
